# In this Issue

**Published:** 2007

## THE GENETICS OF ALCOHOL METABOLISM: ROLE OF ALCOHOL DEHYDROGENASE AND ALDEHYDE DEHYDROGENASE VARIANTS

Alcohol is metabolized by several pathways, the most common of which involves two key enzymes—alcohol dehydrogenase (ADH) and aldehyde dehydrogenase (ALDH). Genetic differences in these enzymes may help to explain why some groups of people have higher or lower rates of alcohol-related problems. For example, certain variations in the genes that produce ADH and ADH have been shown to have a protective effect in that they lead to an increased production of acetaldehyde, a toxic byproduct of alcohol metabolism that can cause adverse physical reactions, such as facial flushing, nausea, and rapid heart beat. This article by Dr. Howard J. Edenberg examines the role ADH and ALDH variants play in alcohol metabolism and the risk for alcoholism. This article also discusses the correlation between occurrence of these genes and alcoholism risk in various ethnic populations. (pp. 5–13)

## VARIATIONS IN ADH AND ALDH IN SOUTHWEST CALIFORNIA INDIANS

Native Americans and Alaskan Natives are five times more likely than other ethnicities in the United States to die of alcohol-related causes. Native Americans are predisposed to alcoholism because of differences in the way they metabolize alcohol. In this article, Dr. Cindy L. Ehlers examines studies that test this hypothesis. Individuals can be protected against or predisposed to alcoholism by variations in the enzymes that metabolize alcohol (i.e., alcohol dehydrogenase [ADH] and aldehyde dehydrogenase [ALDH]). Dr. Ehlers examines the frequency with which these variants occur in one particular group of Native Americans, the Southwest California Indians. The findings suggest that it is unlikely that Native Americans carry a genetic variant that predisposes them to alcoholism. Certain variants of ADH and ADLH do have a protective affect against alcoholism in some Native American people; however, these findings do not explain the high incidence of alcoholism in the tribes that were studied. (pp. 14–17)

## HEALTH-RELATED EFFECTS OF GENETIC VARIATIONS OF ALCOHOL-METABOLIZING ENZYMES IN AFRICAN AMERICANS

The way alcohol is metabolized by the body not only influences drinking behavior but also may play a role in the development of alcohol dependence and alcohol-induced organ damage. Two key alcohol-metabolizing enzymes—alcohol dehydrogenase (ADH) and aldehyde dehydrogenase (ALDH)—and their variants have been shown to influence the risk of alcohol dependence because they mediate the production of acetaldehyde, the toxic byproduct of alcohol metabolism that causes the adverse effects of alcohol consumption. Previous studies have determined that the prevalence of certain variants of ADH and ALDH varies in different ethnic populations. In this article, Drs. Denise M. Scott and Robert E. Taylor examine the prevalence and effects of genetic variants of ADH and ALDH genes in African Americans. For example, one of the *ADH1B* gene variants has been found in up to one-fourth of the people of African descent. This variant results in a higher rate of alcohol metabolism and has been associated with a reduced likelihood of a family history of alcoholism, less positive response to alcohol, and protection against alcohol-related birth defects. (pp. 18–21)

## *ALDH2, ADH1B,* AND *ADH1C* GENOTYPES IN ASIANS: A LITERATURE REVIEW

Previous studies have shown that the prevalence of certain variations of genes for the alcohol-metabolizing enzymes alcohol dehydrogenase (ADH) and aldehyde dehydrogenase (ALDH) can vary across Asian ethnic groups and may cause some groups to have higher rates of alcohol dependence than others. For example, relatively high rates of alcohol dependence have been determined among Koreans and Korean Americans, whereas relatively low rates have been found in Chinese and Chinese Americans. In this article, Drs. Mimy Y. Eng, Susan E. Luczak, and Tamara L. Wall discuss the prevalence of three gene variants—*ALDH2, ADH1B,* and *ADH1C—*among Asian ethnic groups. (pp. 22–27)

## VARIATIONS IN ALCOHOL-METABOLIZING ENZYMES IN PEOPLE OF EAST INDIAN AND AFRICAN DESCENT FROM TRINIDAD AND TOBAGO

On Trinidad and Tobago, differences in alcoholism rates exist among people of East Indian (Indo-Trinidadian) and African (Afro-Trinidadian) ancestry. Researchers have investigated whether these differences can be explained in part by variations in the genes that produce the alcohol-metabolizing enzymes alcohol dehydrogenase (ADH) 1B and 1C and aldehyde dehydrogenase (ALDH) 1 and 2 and cytochrome P450 2E1 (CYP2E1). In this article by Ms. Shelley Moore and Drs. L.K. Montane-Jaime, Lucinda G. Carr, and Cindy L. Ehlers, the authors discuss studies of ADH and ALDH genetic differences in Trinidadians. These studies highlight the usefulness of evaluating risk and protective factors associated with alcohol metabolism in diverse ethnic groups. (pp. 28–30)

## ALCOHOL METABOLISM AND CANCER RISK

Chronic alcohol consumption increases the risk for cancer of the organs and tissues of the respiratory and upper digestive tract, liver, colon, rectum, and breast. Various factors contribute to the development of alcohol-associated cancer, including the effects of acetaldehyde, the toxic byproduct of alcohol metabolism. Alcohol dehydrogenase (ADH) and aldehyde dehydrogenase (ALDH), which are encoded by multiple genes and exist in several variants, are key enzymes involved in alcohol and acetaldehyde metabolism. Because certain variants may result in elevated acetaldehyde levels, the presence of these variants may predispose individuals to certain cancers. Moreover, highly reactive, oxygen-containing molecules (reactive oxygen species) that are generated during certain pathways of alcohol metabolism can damage the DNA and induce tumor development. This article by Drs. Helmut K. Seitz and Peter Becker examines the role of alcohol metabolism in alcohol-associated cancer development, focusing mainly on the contribution of acetaldehyde and on genetic risk factors leading to increased acetaldehyde levels. (pp. 38–47)

## ROLE OF ALCOHOL METABOLISM IN CHRONIC PANCREATITIS

Alcohol abuse is the major cause of chronic inflammation of the pancreas (i.e., pancreatitis). It has been believed that alcoholic pancreatitis is a chronic disease, but recent findings have shown that it may be caused by frequent acute tissue death and inflammation. In this article, Drs. Alain Vonlaufen, Jeremy S. Wilson, Romano C. Pirola, and Minoti V. Apte, discuss the type of pancreas cell that produces digestive juices (i.e., acinar cell) and how alcohol exerts toxic effects on these cells. In addition, there now is sufficient evidence that the pancreas has the capacity to metabolize alcohol via both oxidative and nonoxidative pathways. The resulting metabolites and their byproducts also exert a toxic effect on the pancreas. (pp. 48–54)

## EFFECTS OF PREGNANCY AND NUTRITIONAL STATUS ON ALCOHOL METABOLISM

Fetal Alcohol Spectrum Disorder (FASD) is a constellation of physical, behavioral, and cognitive abnormalities that can result when a fetus is exposed to alcohol in utero. However, only a small percentage of children exposed to alcohol during development display symptoms of FASD, and the mechanisms by which FASD develops are unknown. In this article, Drs. Kartik Shankar, Martin J.J. Ronis, and Thomas M. Badger speculate that nutrition and alcohol exposure may interact to contribute to the development of FASD. Because undernutrition can slow the rate of alcohol metabolism, and exposure to alcohol may contribute to under-nutrition, it is difficult to determine the precise effects of these factors. However, the researchers suggest that improving maternal nutrition during pregnancy, which minimizes fetal exposure to alcohol, might reduce the incidence of FASD among high-risk populations. (pp. 55–59)

